# Supplementary material for: Structure of HK97 small terminase:DNA complex unveils a novel DNA binding mechanism by a circular protein
Source: bioRxiv. 2023 Jul 20:2023.07.17.549218. Preprint. [Version 2] doi: 10.1101/2023.07.17.549218 (PMC10370121; doi:10.1101/2023.07.17.549218)
Supplement: Supplement 1 [file media-1.pdf]

Supplementary information

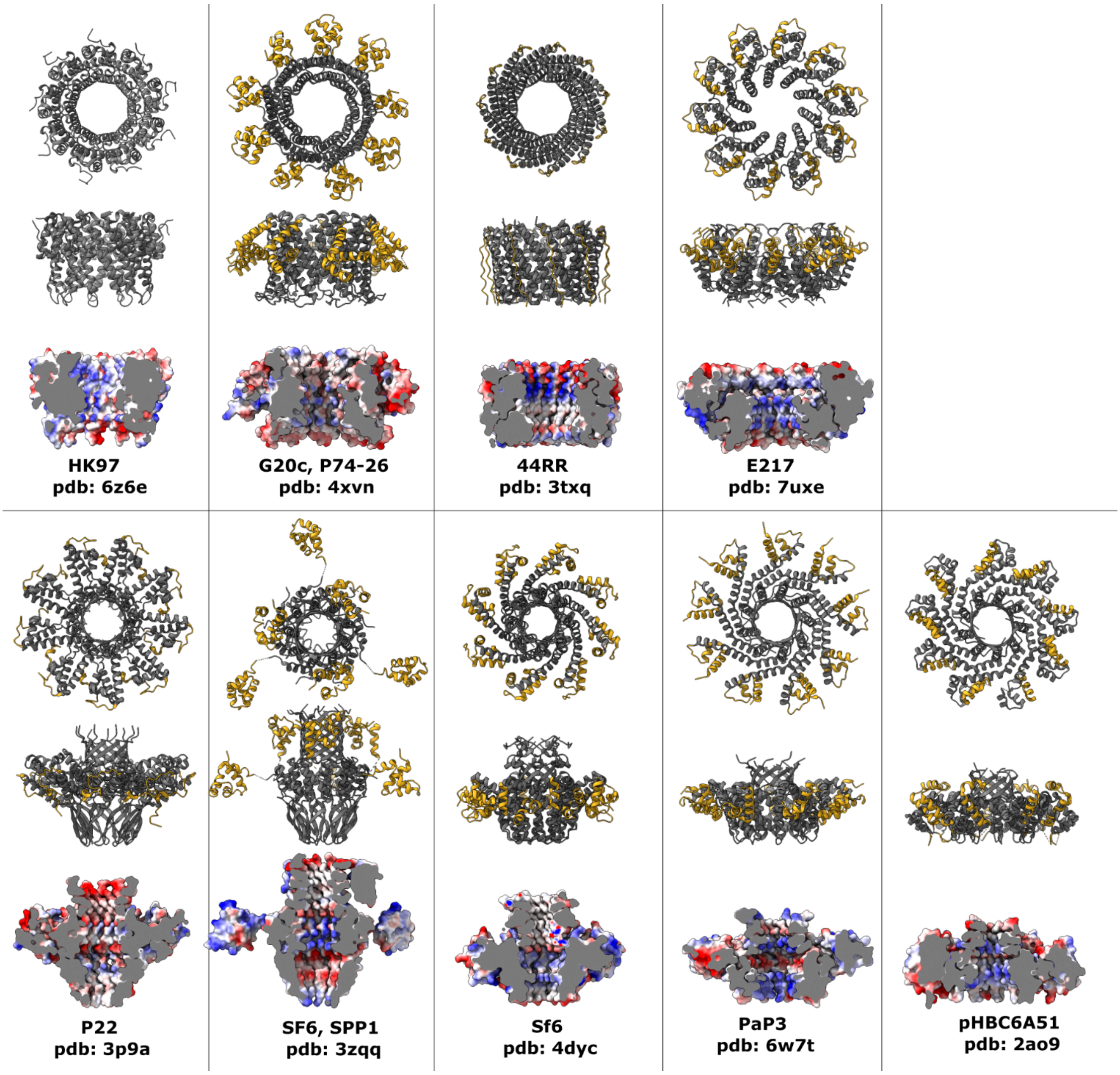

Supplementary Figure 1. Structures of small terminases deposited in PDB and EMDB. All structures are shown at the same scale: top and side view are presented with oligomerisation domain and C-terminus in grey and DNA binding domain (confirmed experimentally or predicted) in gold. Below is the cross section of the same structure coloured by electrostatic potential (red - negatively charged, blue - positively charged, range -16.7 to 16.7 kT/e).

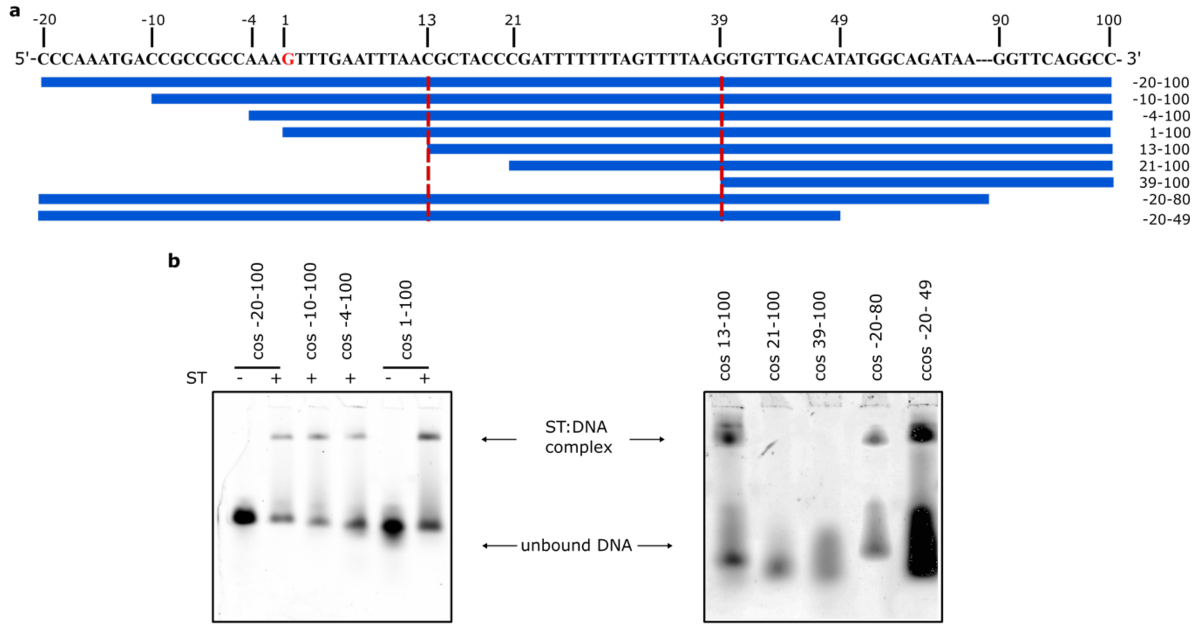

Supplementary Figure 2. Determination of small terminase binding site. a Sequence of HK97 putative small terminase binding site showing oligos used. Position 1 - cleavage site during genome packaging. The minimal small terminase binding site is marked with red dotted lines. **b** EMSA of interaction of fluorescent oligos spanning different regions of the putative binding site with small terminase.

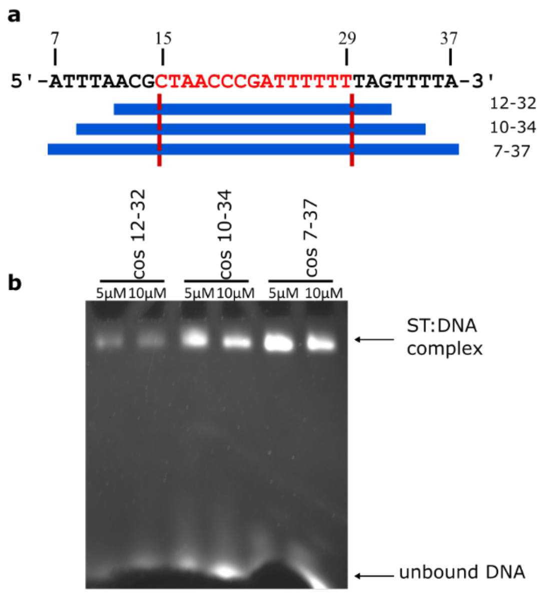

Supplementary Figure 3. Oligo optimisation for small terminase:DNA complex formation. a Sequence of small terminase binding site showing oligos used. **b** EMSA of the interaction of these oligos with small terminase.

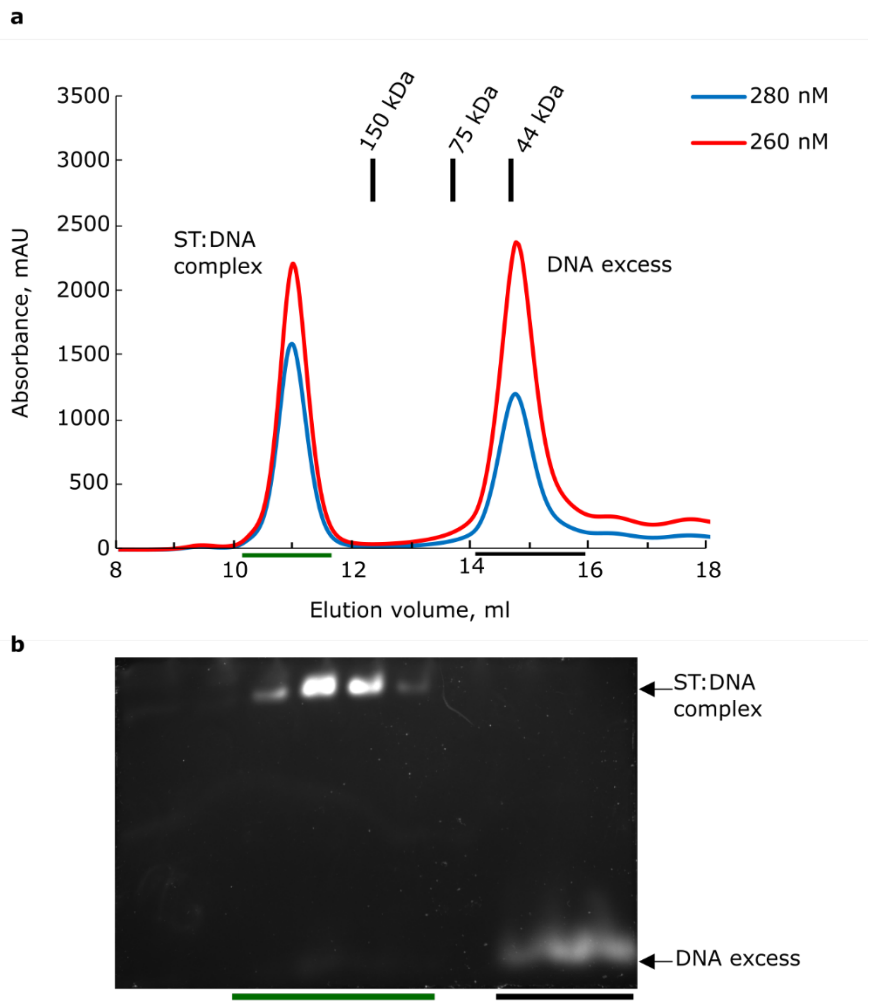

Supplementary Figure 4. Purification of small terminase:DNA complex on S200 10/30 column. a Analytical size exclusion chromatography profile. **b** Native SDS gel stained with EtBr, with elution fractions for the complex (green bar) and excess of DNA (black bar) labelled.

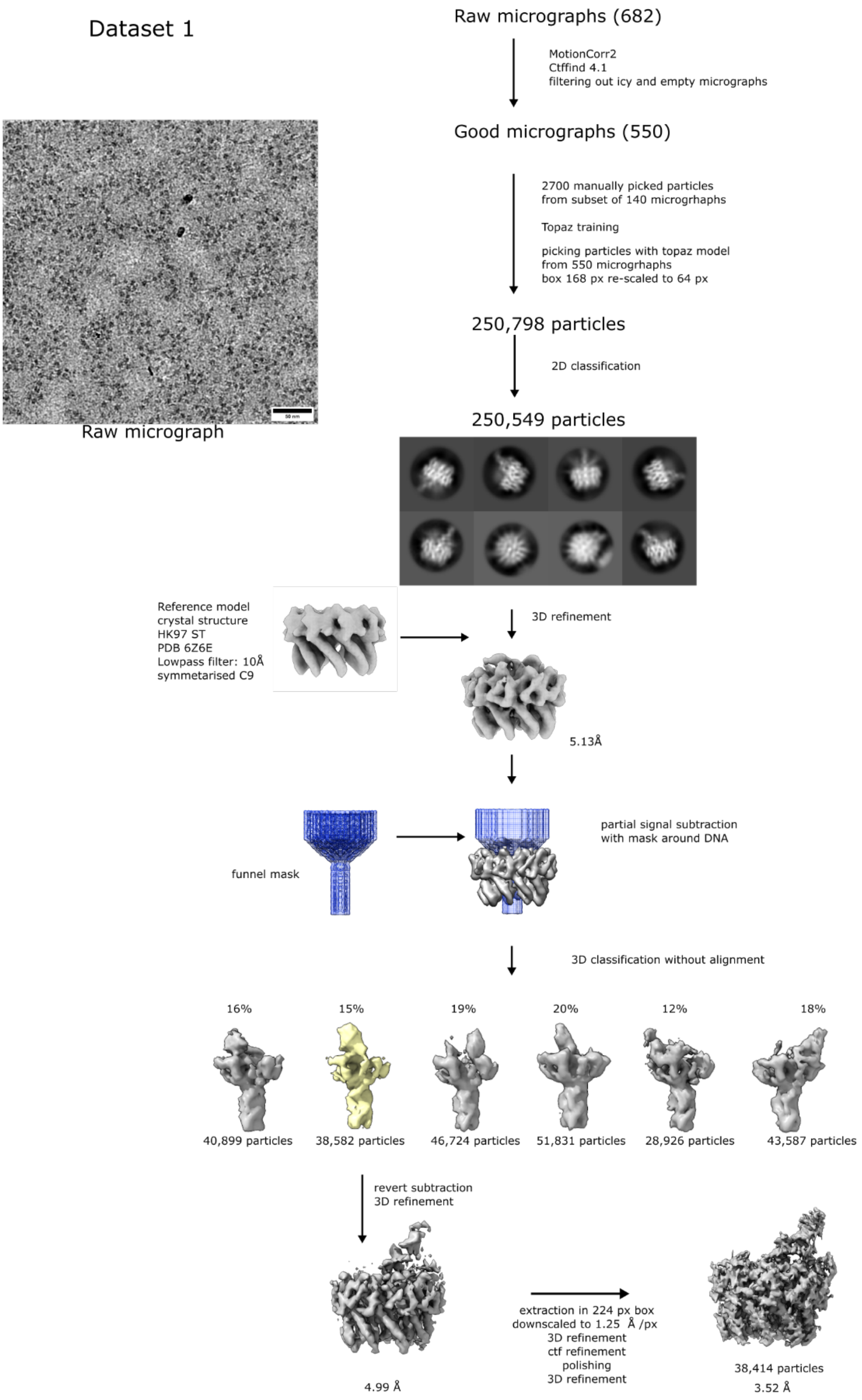

Supplementary Figure 5. **CryoEM processing flow chart for dataset 1.** Overview of initial cryoEM processing steps for small terminase:DNA complex dataset 1. Mask used in focused classification with partial signal subtraction is shown in blue. A representative micrograph showing particle distribution (scale bar is 50 nm) and a selection of 2D class averages representing different particle views is shown.

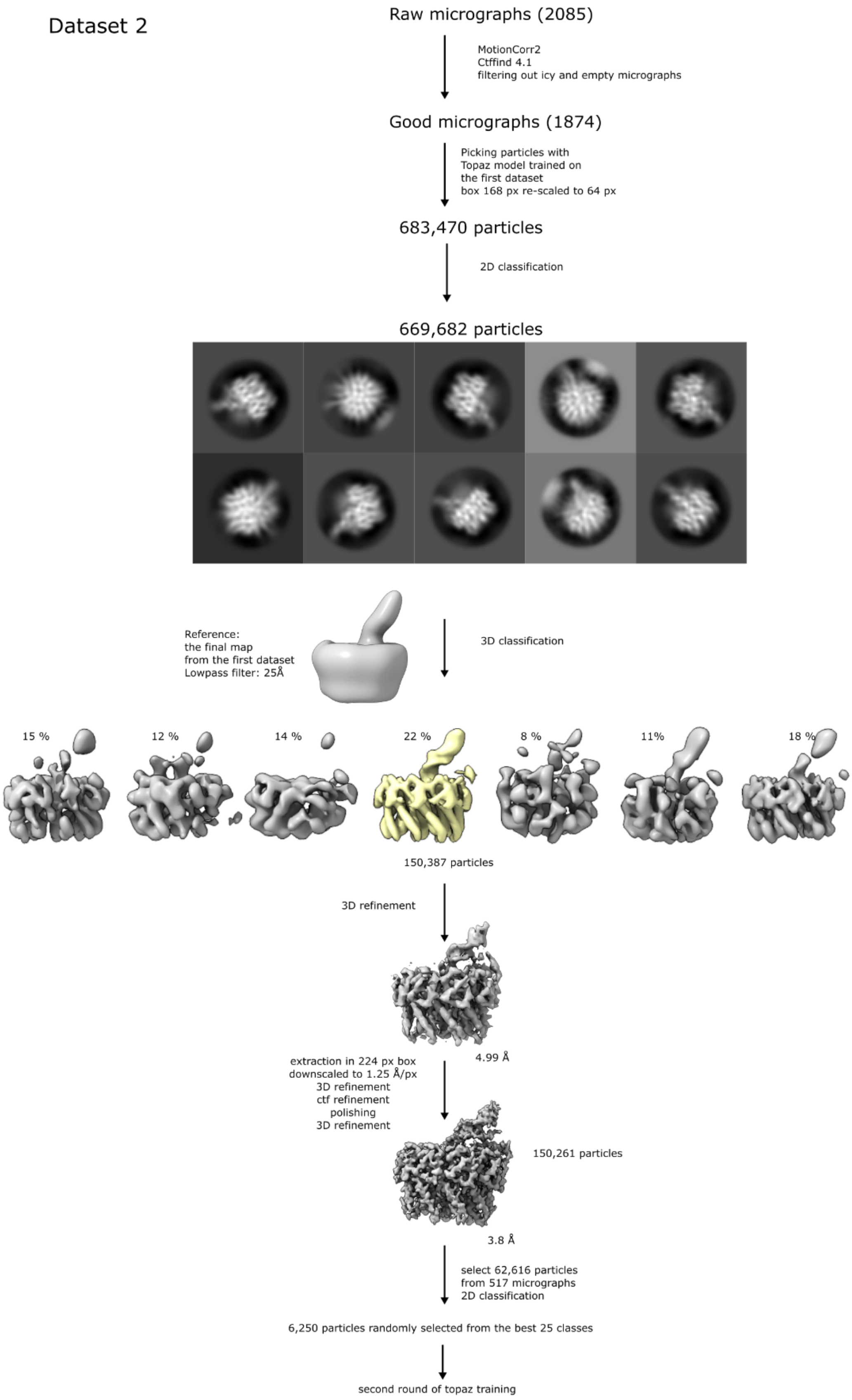

Supplementary Figure 6. **CryoEM processing flow chart for dataset 2.** Overview of the initial cryoEM processing steps for small terminase:DNA complex dataset 2. A selection of 2D class averages representing different particle views is shown.

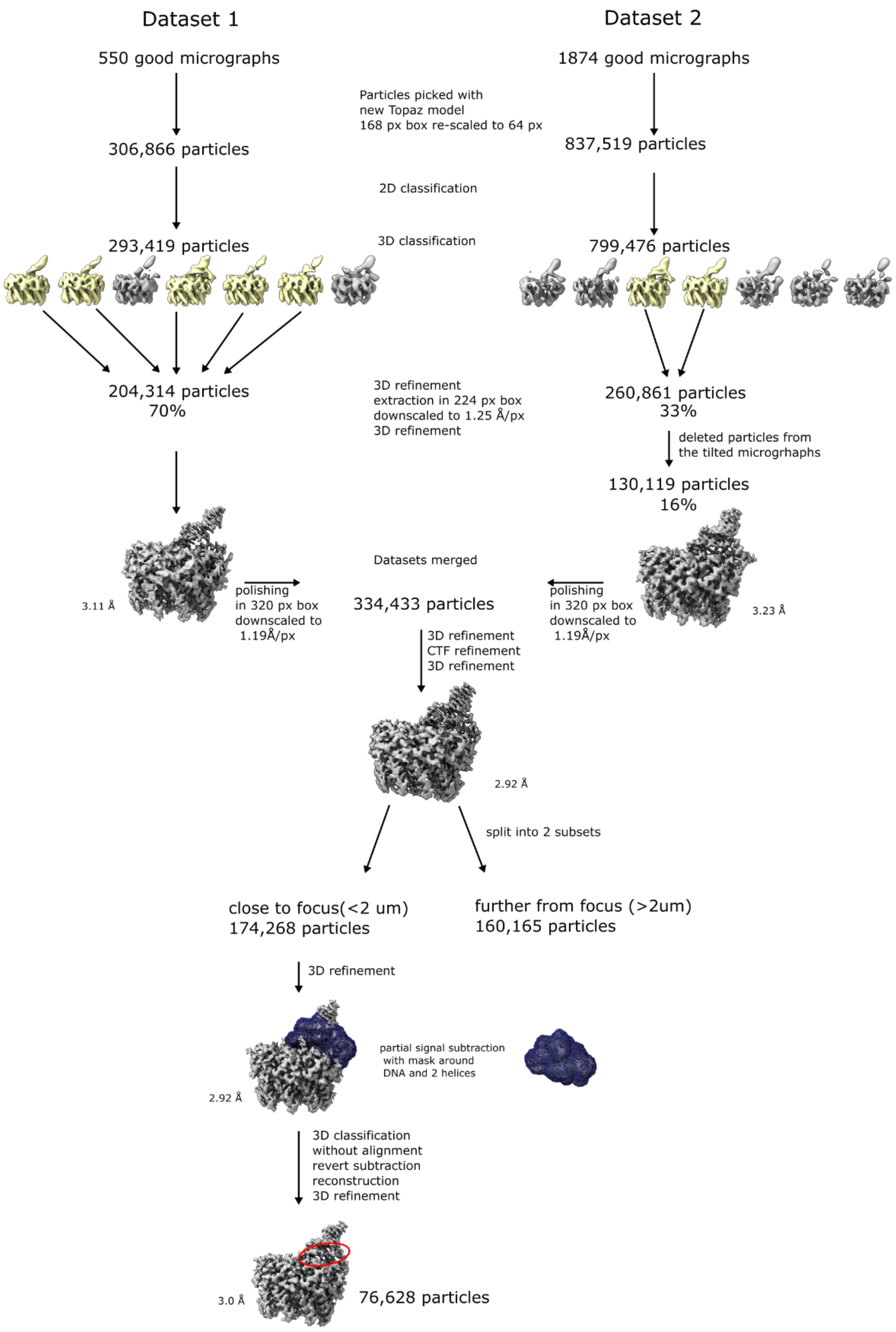

Supplementary Figure 7. **Final CryoEM processing flow chart for merged data from both datasets.** Overview of the cryoEM processing steps for the combined datasets. Particles were picked with a Topaz model trained with the "good particles" from the final model for dataset 2. Mask for focused classification with partial signal subtraction around two helices and a region of DNA is shown in blue. Red oval highlights the area of the structure where resolution was improved after focused 3D classification.

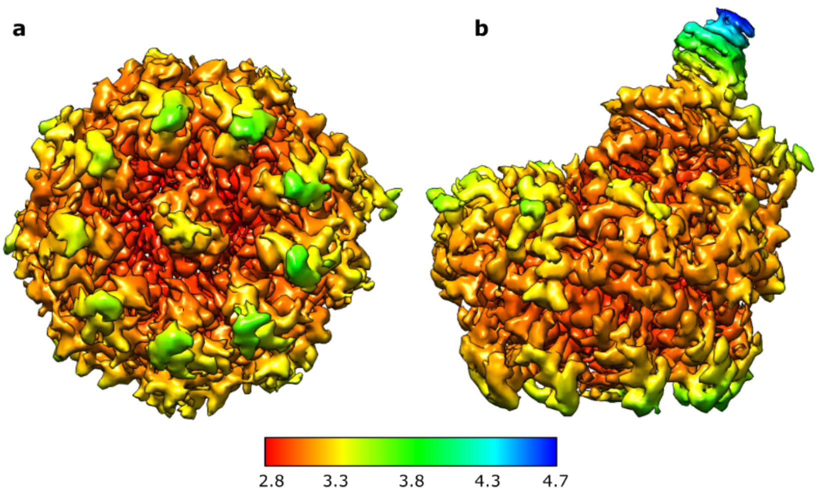

Supplementary Figure 8. CryoEM map coloured by local resolution. **a** Bottom view**. b** Side view.

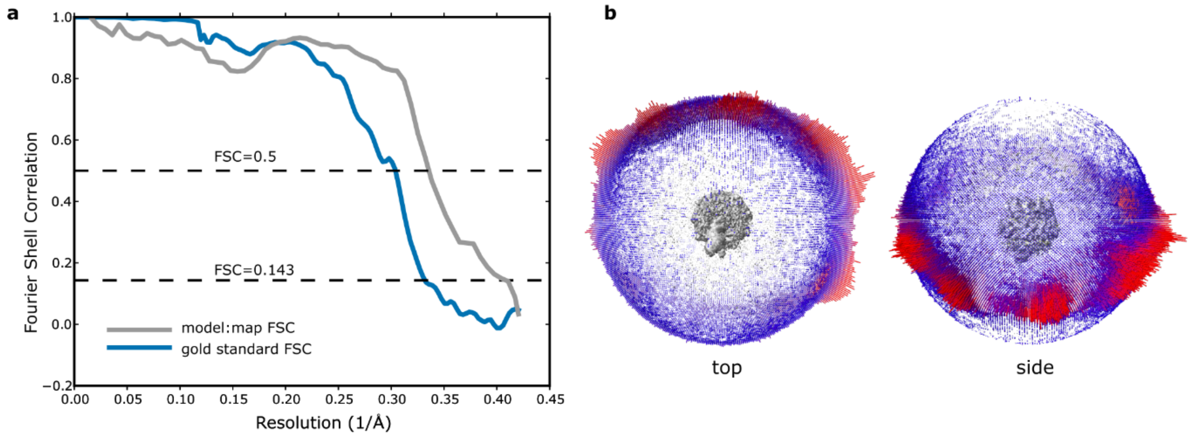

Supplementary Figure 9. a ‘Gold standard’ FSC curve (blue) and model:map FSC curve (grey). Dashed lines indicate FSC cut-offs of 0.143 and 0.5 for ‘gold standard and model:map FSC, respectively. **b** Euler angle distribution of particles contributing to the cryoEM reconstruction.

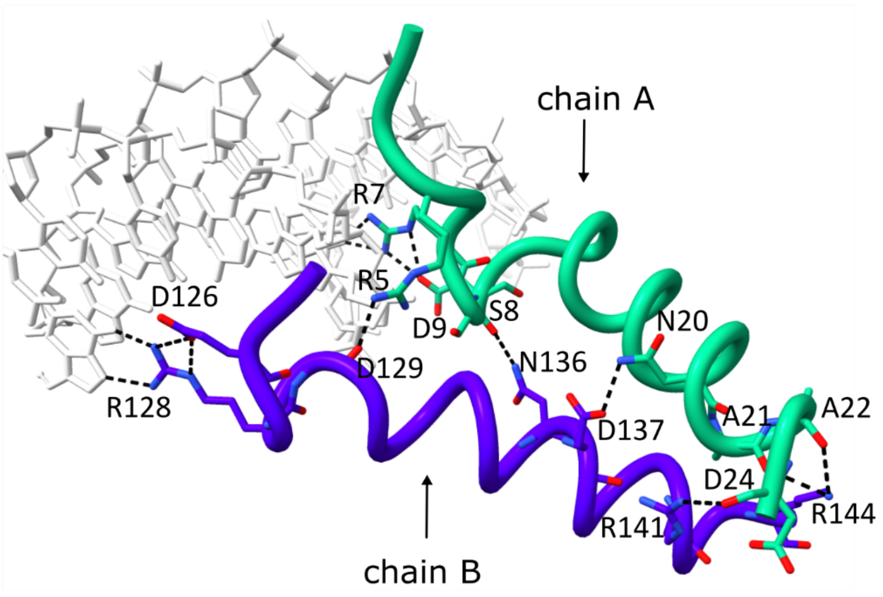

Supplementary Figure 10. DNA binding substructure. N-terminus of chain A is shown in green**,** C-terminus of chain B is in purple, DNA is in white, hydrogen bonds are depicted as black dashed lines.

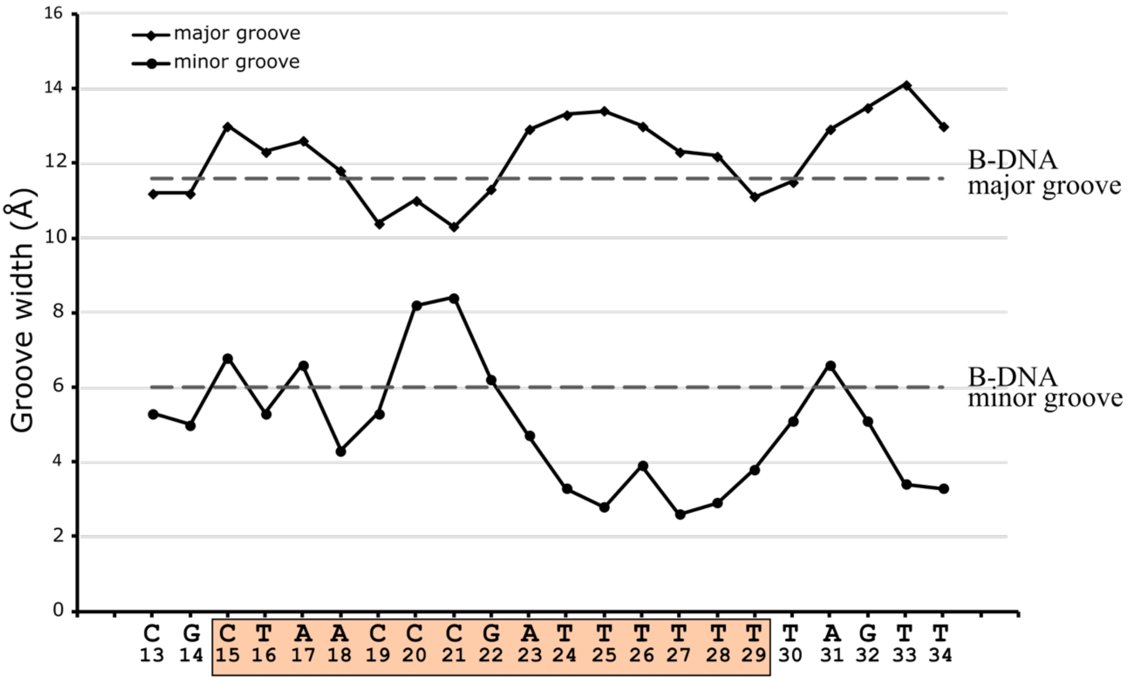

Supplementary Figure 11. Major and minor groove width. The widths of major and minor groove in DNA in the small terminase:DNA complex calculated using the program CURVES+ (Lavery & Sklenar, 1989). The average major and minor groove widths of B-form DNA (Chandrasekaran & Arnott 1996) are shown as a dotted line. Small terminase binding site is highlighted in orange.

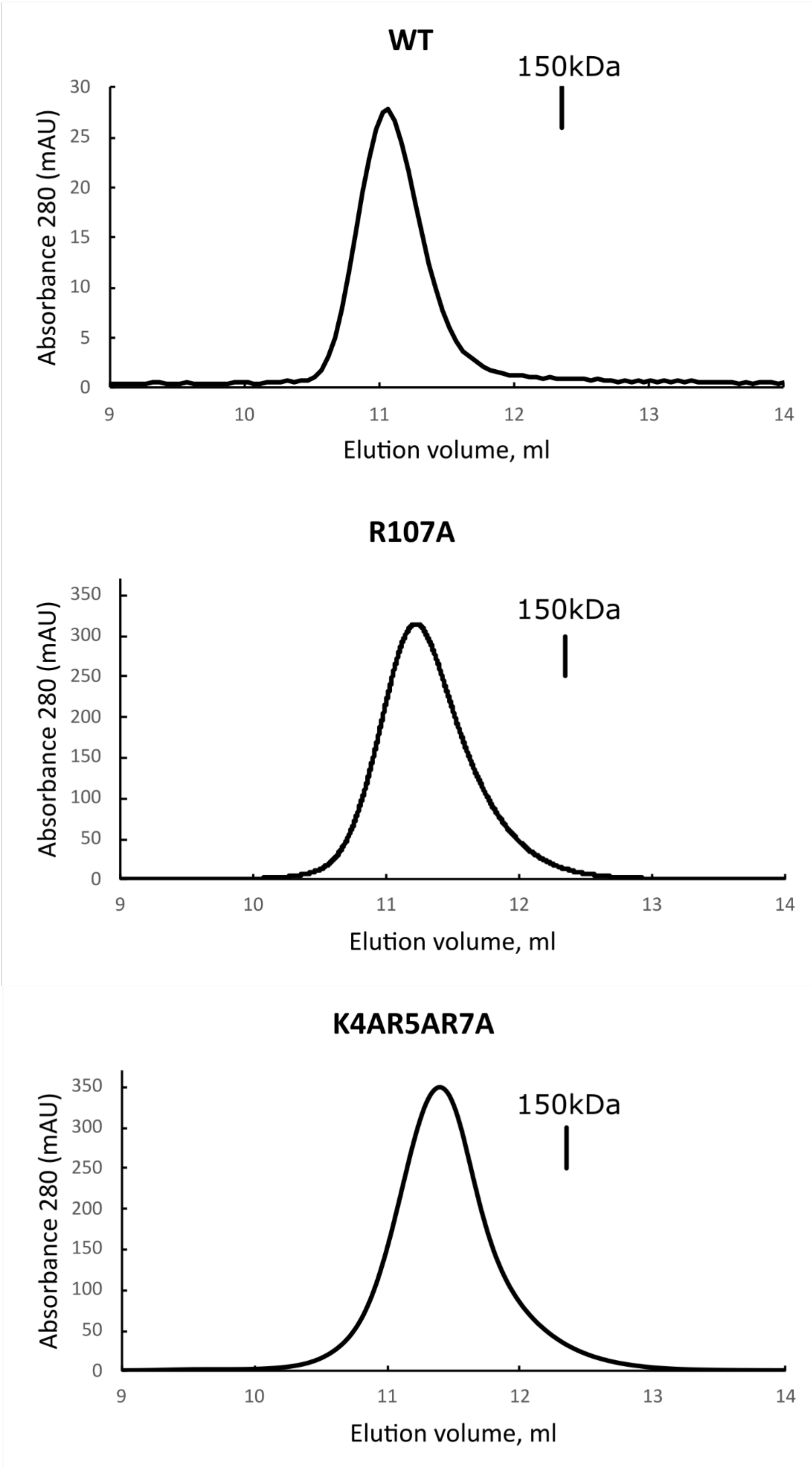

Supplementary Figure 12. Purification of small terminase mutants. Elution profiles of wild type (WT) protein and mutants R107A and K4AR5AR7A on S200 10/30 column.

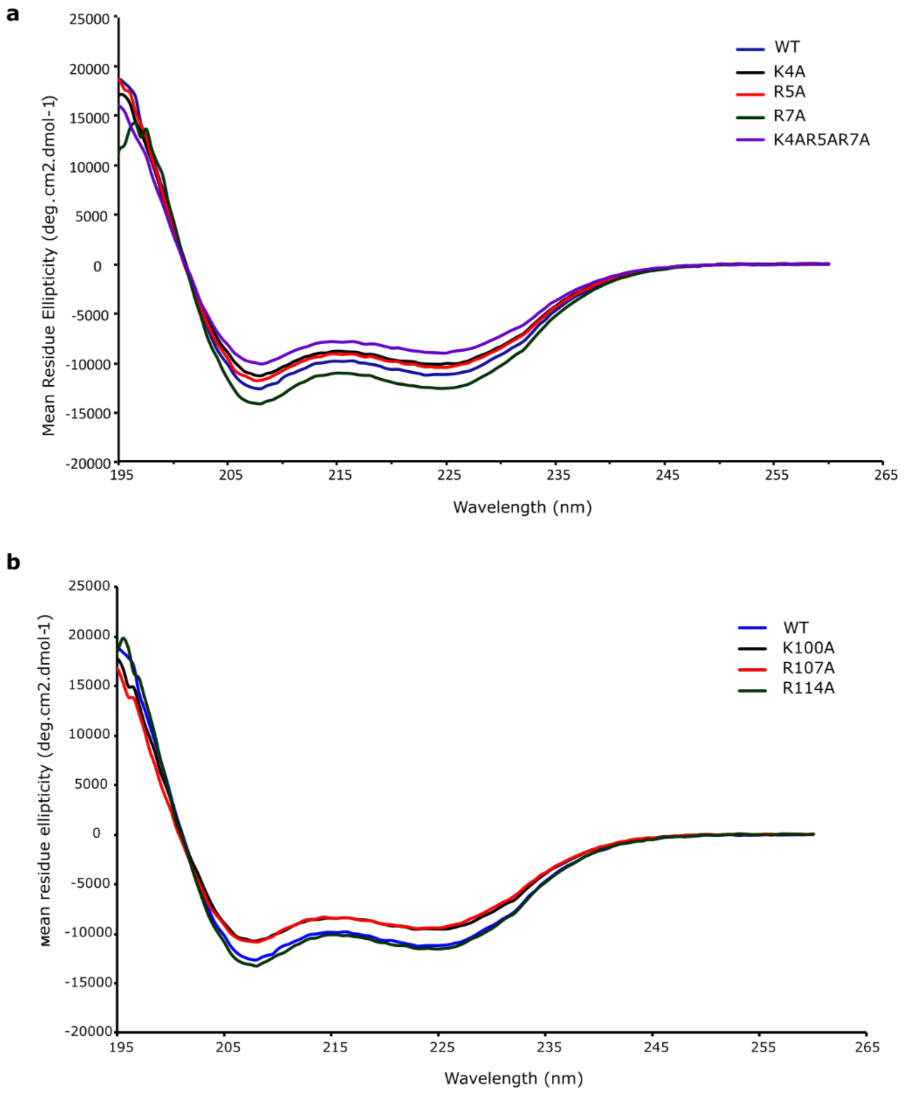

Supplementary Figure 13. Far-UV circular dichroism spectra of wild-type (WT) and mutants of small terminase. **a** Mutants of N-terminal arm. **b** Mutants within the channel of small terminase.

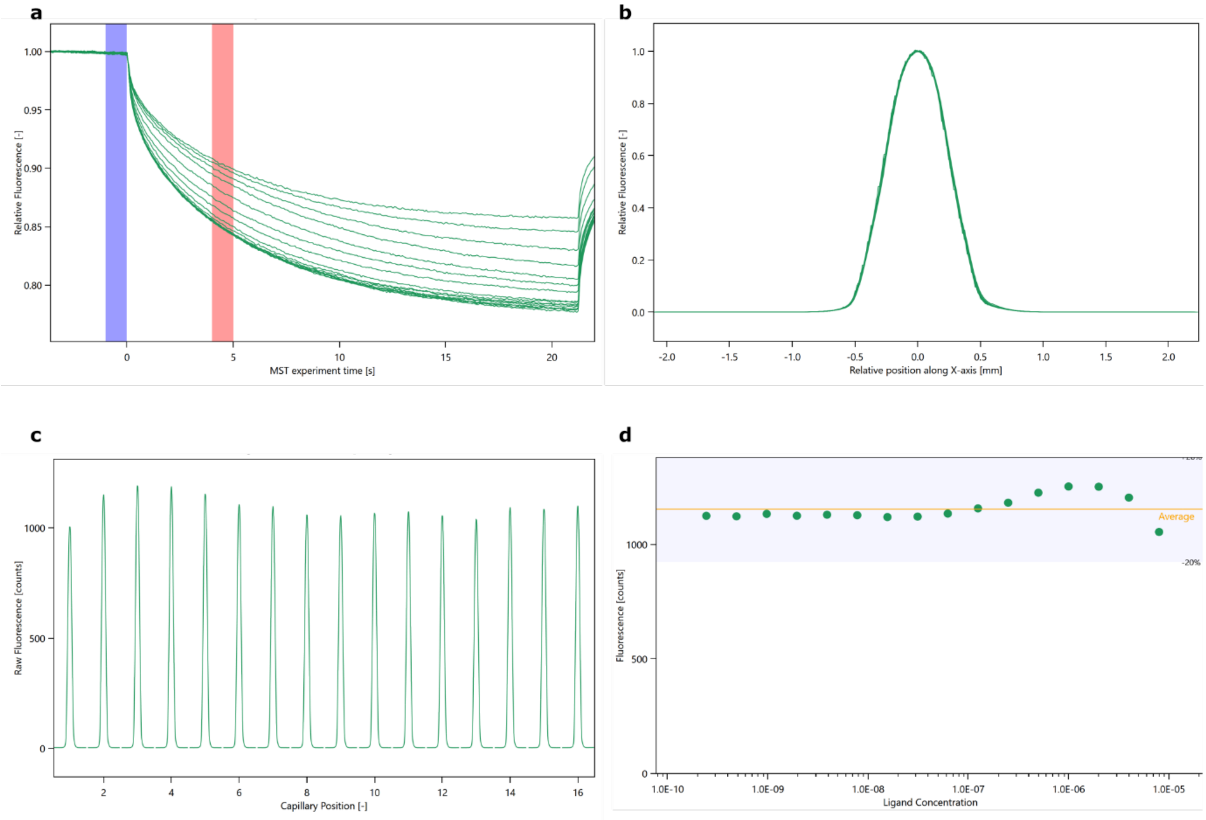

Supplementary Figure 14. MST original data for one run of WT small terminase. **a** Fluorescent traces over time for each capillary containing different concentrations of protein **b** Representative scan of fluorescence intensity across one capillary. **c** Representative capillary scan of fluorescent intensity across all capillaries. **d** Plot showing initial fluorescence intensity across all samples (capillaries) prior to temperature jump.

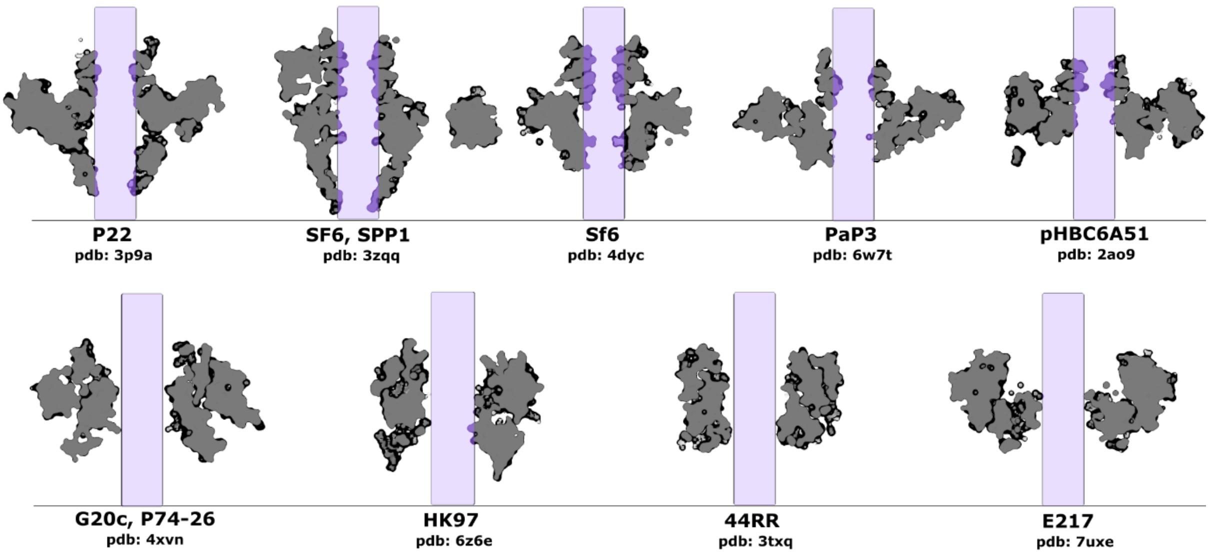

Supplementary Figure 15. Comparison of the central channel of small terminases. 3 Å thick section through the middle of small terminase models with 20 Å diameter cylinder positioned along the channel axis.

Supplementary Table 1. Statistics for cryoEM data collection and processing.

Data collection	Dataset 1	Dataset 2
Microscope/detector	Krios/Gatan K2Summit with energy filter (slit width of 20 eV)	
Voltage (kV)	300	
Nominal magnification	130,000x	
Recording mode	counting	
Flux (e- /Å^2/s)	6.65	6.7
Target defocus (µm)	1.3 to 3.1	1 to 2.4
Pixel size Å	1.07	1.07
Fluence (e- /Å^2)	53	54
Number of fractions	50	50
Total exposure time (s)	8	8
Number of movies	682	2085
Total particles picked	306866	837519
Particles from both datasets used in final reconstruction	334433	
Map resolution at FSC = 0.143 (Å)	2.9	
Local resolution (Å)	2.9-4.9	
Map sharpening B-factor (Å^2)	-49.9	
Particles after focused classification with partial signal subtraction	76626	
Map resolution at FSC=0.143 (Å)	3.0	
Map sharpening B factor (Å^2)	-30.9	

Supplementary Table2. Refinement statistics and validation.

Map resolution range refined against (Å)	3.0 – 114.1
Non-hydrogen atoms	
Protein	7784
Nucleic acid	1145
Average B factors (Å^2)	
Protein	87.5
Nucleic acid	115.5
R.m.s. deviations	
Bond lengths (Å)	0.0059
Angles (°)	1.335
Validation	
MolProbity score	1.11
Clashscore	3.16
Rotamer outliers (%)	0.12
Ramachandran plot	
Favoured (%)	99.25
Outliers (%)	0.0

Supplementary Table 3. N- termini sequences of DNA binding proteins with N-terminal arm (NTA) and all known small terminases. Grey boxes- not modelled residues, light blue boxes – NTA, red font - positively charged residues, yellow boxes – non structured residues, green boxes – helices, purple box – β-sheet.

PDB	Protein/phage	Sequence
5ZJQ	Homeobox extradenticle chain A	KKRKPYSKFQTLELEKEF
5ZJQ	Homeobox abdominal-B chain B	DARRKRRNFSKQASEILNEYFYS
1W0T	hTRF1	KRQAWLWEEDKNLRSGVRKYG
1W0U	hTRF2	KKQKWTVEESEWVKAG
HK97	Enterobacteria phage HK97	MADKRIRSDSSAAAVQAMKNAA
4Z3C	Bacillus phage SF6	MKEPKLSPKQERFIEEYFIN
3HEF	Enterobacteria phage Sf6	MATEPKAGRPSDYMPEVADDICSLLSS
3P9A	Enterobacteria P22	MAAPKGNRFWEARSSHGRNPKFESPEALWAAC
2A09	Bacillus cereus	MPFSISGRKGSEMMAKLDELKQKLTAK
3TXQ	44RR	MNDVLDFTQLKDLNGIEGIHGEDVQVYAPLVLRDPVSNPNNRKIDQDDDYELVRRN
4XNV	Thermus phage G20c	MSVSFRDRVLKLYLLGF
6W7T	Pseudomonas virus PaP3	MSDEKVVSIGAAPLSAKEKLDLYCE
7UXE	Pseudomonas E217	MTKFYSPDDLVTPQEFADPHFAAINQKRFDLYIDLRVQG
1J9I	Escherichia phage lambda	MEVNKKQLADIF

Supplementary Table 4. Borders of DNA binding domains or N-terminus regions highlighted in gold in Supplementary Figure 1.

Phage	PDB	
P22	3p9a	n-terminus: 4-24 aa
SP6	3zqq	HTH: 10-60 aa
Sf6	4dyc	HTH: 10-52 aa
PaP3	6w7t	HTH: 13-46 aa
pHBC6A51	2ao9	HTH: 23-48 aa
G20c	4xvn	HTH: 1-52 aa
HK97	xxx	DBM: 3-24 aa chain A, 125-145 aa chain B
44RR	3txq	n-terminus: 25-40 aa
E217	7uxe	HTH: 14-50 aa

References:

R. Lavery and H. Sklenar (1989) "Defining the structure of irregular nucleic acids: conventions and principles." J Biomol Struct Dyn Vol. 6 Issue 4 Pages 655-67

R. Chandrasekaran and S. Arnott, (1996) "The structure of B-DNA in oriented fibers." J Biomol Struct Dyn Vol. 13 Issue 6 Pages 1015-27
